# Supplementary material for: Molecular characterization of Blastocystis subtypes in symptomatic patients from the southern region of Syria
Source: PLoS One. 2023 Mar 16;18(3):e0283291. doi: 10.1371/journal.pone.0283291 (PMC10019620; doi:10.1371/journal.pone.0283291)
Supplement: Attachment — Submitted filename: Reviewer report.pdf [file pone.0283291.s001.pdf]

MANUSCRIPT ID: PONE-D-22-27237

TITLE: Molecular characterization of Blastocystis subtypes in symptomatic patients from the southern region of Syria

GENERAL

This manuscript describes the occurrence and distribution of different Blastocystis subtypes detected in 48 samples out of 60 samples of symptomatic patients from southern region of Syria. The distribution was compared according to age, gender, presence of domestic animals and availability of water supply. Moreover, association of different subtypes (single or mixed) with clinical symptoms was investigated. Five subtypes were identified; with ST1 was the predominant subtype followed by ST3.

Overall, the manuscript is straightforward; the findings are interesting and provide baseline molecular information on the distribution of Blastocystis subtypes in Syria. However, major drawbacks that clearly appear in the study design, data analysis and results presentation should be adequately addressed before this manuscript can be acceptable for publication. Please refer to the comments given below.

MAJOR REVISION

1. Justification of the study should be strengthened. If this is the first study to provide molecular information about Blastocystis in Syria; then this should be clearly stated as a justification; in addition to some epidemiological characteristics of the study area or some population-specific characteristics. If previous studies from other regions of Syria are available then related findings should be presented and research gaps should be stated.

2. The manuscript lacks information about the study design. Information about study design should be added to abstract and methods section. A STROBE checklist, used for the reporting of observational studies, can be downloaded (http://www.strobe-statement.org/index.php?id=available-checklists), filled in applicable manuscript section, and then the completed checklist can be uploaded as a Supporting Information file.

3. The manuscript lacks important information about the study area. What is the name of included city (cities) or districts? Was it a hospital-based or community-based study? What are the characteristics related to intestinal parasites? E.g. sanitation, rural or urban or semi-urban settings, etc. Such information is important for findings generalizability.

4. The manuscript lacks important information about the study population. Information on the recruitment strategy, sample size estimation, sample collection and transport should be provided. Such information is important for findings generalizability.

5. The sample size is small (n = 60). This should be acknowledged as a limitation. Another limitation should also be acknowledged; bacterial and viral causes of symptoms were not ruled out.

6. Data analysis and findings presentation should be improved. Refer to comments # 15-17.

MINOR REVISION

7. Introduction, 2nd sentence: Indeed, pathogenicity of Blastocystis is still controversial. This should be clearly indicated.

8. Line 68: "…from residing patients of the southern regions of Syria…". What were the included cities or districts or health centers?

9. Line 76: cite a reference for the adopted diagnostic criterion.

10. Line 113: "assess the correlation" change to "assess the association".

11. Line 122: rephrase the sentence. The word "isolates" can be a source of confusion to readers.

12. Line 124: what about the results of the other 12 patients? Was there any parasites detected or they were negative for any parasites?

13. Line 128: "60 isolates". Does this refer to the 60 samples or the overall detected subtypes in the 48 samples?

14. Line 137-138: add the percentage after the subtype; i.e. by ST1+2 (5%) and ….

15. Table 2: It can be modified to improve clarity. Remove first total (%) row; and add its data to Single infection row. Then, add a row for co-infection with the data of last row. Then, add a row of Type of co-infection with the 5 rows of combination.

16. Table 4: it should be split into 2 tables. Table 1 for associated factors. Because of small number of positive cases, this table can include all samples; add a column for N examined (n=60) then a column for overall Blastocystis infection, then a column for P. Add columns for ST1 and ST3 with P column for each. The ST1 data should include all ST1 (single and mixed and co-infection; i.e. 34 cases (18+11+5), ST3 should include all ST3 (7+6+1!). Chi square test is not applicable for all variables, indicate where Fischer's Exact test used. The table design can be as follow:

Variable	Examined	Overall infected n (%)	P	ST1 n (%)	P	ST2 n (%)	P	Mixed STs n (%)	P
Overall n	60	48		34		14!		?	
Gender									
Male									
female									

17. Table 4: Revise variables grouping. Age groups can be 4 – 20 and the last one should be > 40. Presence of animals should be Presence of domestic animals (Yes or No), why "Presence of insects" is mentioned here?

18. The new table 5 can be for Association of common subtypes and clinical symptoms. Modify the table to avoid repeating Chi square/p value rows. This table should include

single infections of related subtypes, as it is. So, add columns for P values after each subtype column. No need to report Chi square values, just the P values and indicate the test used in footnote.

19. Lines 163-165: But these are for ST1 only, as indicated by the p value.

20. Avoid repeating results in discussion.

21. Are there previous findings from other regions of Syria? If yes, compare and discuss. Findings from neighbouring countries (Jordan, Lebanon, Iraq, Turkey, etc) can be used too.

22. Lines 196-198: indicate the limitation here that bacterial and viral causes were not ruled out.
