# Supplementary material for: Molecular characterization of Blastocystis subtypes in symptomatic patients from the southern region of Syria
Source: PLoS One. 2023 Mar 16;18(3):e0283291. doi: 10.1371/journal.pone.0283291 (PMC10019620; doi:10.1371/journal.pone.0283291)
Supplement: Attachment — Submitted filename: Response to Reviewers.docx [file pone.0283291.s002.docx]

**Response to Reviewers**

**Reviewer #1:**

We would like to thank you for your precise and valuable time and comments, please find below our response to each comment.

1. Line 30: rRNA not italicised. Amend here and through the whole manuscript.

**Done**

2- Line 31: 100% (without space).

**Done**
3. Line 50: Please note that four new Blastocystis STs (ST35-ST38) have been recently described. See Maloney et al. Microorganisms. 2022;11(1):46. doi: 10.3390/microorganisms11010046. Please update this information.

**Modification has been done**

4. Line 68: In my previous appraisal, I specifically requested improving this subsection by providing information on inclusion/exclusion criteria followed to recruit patients, defining the clinical manifestations considered, and indicating the hospital/medical centers participating in the study. These requests, which are still pending

**We did not have any exclusion criteria; we only included all patients suffering from different gastrointestinal symptoms.**

**We modified all other requested points.**
